# Micro- and Nanoplastics as a Potential Risk Factor for Stroke: A Systematic Review

**DOI:** 10.3390/jox16010034

**Published:** 2026-02-14

**Authors:** Jakub Kufel, Miłosz Korbaś, Julita Janiec, Zofia Pankowska, Marta Młynek, Aleksandra Gaweł, Adam Mitręga

**Affiliations:** 1 Department of Radiology and Nuclear Medicine, Faculty of Medical Sciences in Katowice, Medical University of Silesia, 40-752 Katowice, Poland; 2 Students’ Scientific Association of Computer Analysis and Artificial Intelligence, Department of Radiology and Nuclear Medicine, Medical University of Silesia, 40-055 Katowice, Poland; 3 Department of Biophysics, Faculty of Medical Sciences in Zabrze, Medical University of Silesia, 41-808 Zabrze, Poland

**Keywords:** nanoplastics, microplastics, stroke, cerebrovascular disease, cardiovascular risk, environmental pollution

## Abstract

Environmental pollution with micro- and nanoplastics (MNPs) is an escalating global health concern. Despite growing evidence of MNPs’ presence in the human body, their impact on cerebrovascular diseases remains poorly understood. This study aimed to systematically assess the presence of MNPs in the vascular system and their association with the risk and progression of stroke. A systematic review was conducted in accordance with PRISMA 2020 guidelines and registered in PROSPERO (CRD420251272759). PubMed, Scopus, Web of Science, and Embase databases were searched for original research articles published in the last 10 years. Five studies were included (2 human observational, 3 animal in vivo), comprising 287 patients and rodent models. Methodological quality was assessed using ROBINS-E and SYRCLE’s RoB tools. The analysis confirmed the presence of MNPs, particularly polyethylene and polyvinyl chloride, in key human pathological structures, including carotid atherosclerotic plaques and stroke thrombi. Notably, the presence of MNPs in plaques was associated with a 4.5-fold increase in the risk of major cardiovascular events and death. Animal model studies provided a biological rationale for these observations, demonstrating that MNP exposure may lead to microembolization in cerebral circulation, blood–brain barrier disruption, and exacerbated ischemic injury. Importantly, MNP burden may reflect cumulative environmental exposure and vascular disease severity rather than a direct causal factor in stroke pathogenesis. Nevertheless, MNPs may still represent a novel, modifiable risk factor for stroke through their association with adverse vascular outcomes. Available evidence confirms their accumulation in the cardiovascular system and suggests an association with adverse clinical outcomes. Due to the limited number of studies, further standardized research on larger populations is required to establish whether a causal relationship exists.

## 1. Introduction

Stroke remains the second leading cause of death and the third leading cause of combined death and disability globally. In 2021, approximately 11.9 million new strokes and 93.8 million stroke survivors were recorded, with the burden increasing more rapidly in lower-income countries. European direct costs reached 26.6 billion euros, and WSO-Lancet projections estimate a ~50% rise in stroke mortality between 2020 and 2050 [1,2]. Parallel to the growing burden of cerebrovascular and cardiovascular diseases, attention has increasingly focused on environmental contaminants that may contribute to vascular pathology, including micro- and nanoplastics (MNPs). Nowadays, human exposure to MNPs is considered unavoidable due to their ubiquity in the environment. Microplastics (MPs) are detected in drinking water worldwide, with 87% of tap water samples across 34 countries containing them, predominantly as fragments below 50 μm, most commonly transparent polyester particles [3]. Food sources further contribute to daily intake, with MNPs reported in seafood, fish, salt, honey, sugar, vegetables, beverages, and dairy products [4,5]. Inhalation of airborne particles and dermal contact represent additional exposure routes, highlighting the continuous and multi-pathway nature of human contact with microplastics. Accumulating evidence indicates that MNPs may enter systemic circulation and distribute to multiple organs. They have been detected in human carotid atherosclerotic plaques, blood, cardiac tissue, and the brain. Patients with detectable MNPs in excised plaques demonstrated over a fourfold increase in major cardiovascular events, including myocardial infarction, stroke, or death, over a 34-month follow-up compared with individuals without such deposits [6,7]. Additional studies have identified MNPs in feces, placenta, lungs, and other tissues, with inflammatory states facilitating translocation by increasing vascular and epithelial permeability [8,9,10]. Importantly, nanoplastic particles may cross the blood–brain barrier (BBB), reaching the central nervous system [11]. Biological mechanisms linking MNPs to vascular injury have been proposed, including oxidative stress, inflammation, endothelial dysfunction, prothrombotic activity, and direct myocardial damage leading to fibrosis and impaired contractility [8,10,12]. Experimental data also show that MNPs promote microvascular thrombosis in the brain, resulting in ischemia and neurological deficits [13]. Population-level analyses further suggest an association between higher environmental MNP exposure and increased prevalence of cardiovascular disease and stroke in affected regions [14]. Advances in analytical and imaging technologies have substantially improved the detection and characterization of MNPs in biological matrices. Techniques such as Raman spectroscopy, Fourier-transform infrared spectroscopy, scanning and transmission electron microscopy, TED-GC/MS, fluorescence methods, and confocal imaging now allow the identification of increasingly smaller particles across complex tissues and fluids [15,16,17]. These methodological developments enable more precise assessment of polymer composition, size distribution, and particle burden, making systematic synthesis of available findings both feasible and necessary. Despite rapidly expanding evidence on systemic microplastic distribution, no existing review has synthesized data specifically regarding the presence and characteristics of MNPs in adult stroke patients. As a result, critical questions remain unresolved, including the types of polymers detected in cerebrovascular disease, the particle sizes and concentrations involved, their anatomical locations, and the consistency of detection methods across studies. Importantly, MNPs detected in vascular and neural tissues may represent markers of cumulative environmental exposure and disease severity rather than a direct cause of cerebrovascular pathology. Nevertheless, this observation highlights a significant and clinically relevant research gap given the potential for MNPs to act as a novel, modifiable vascular risk factor. The objective of this review is therefore to synthesize and critically appraise the available evidence regarding MNPs in adult stroke patients, with a focus on polymer types, particle sizes, concentrations, anatomical locations, detection techniques, and reporting quality. If excessive heterogeneity is encountered, the synthesis will be limited to a narrative approach, accompanied by a risk-of-bias assessment and the identification of priorities for future research.

## 2. Material and Methods

This study was registered in PROSPERO under the number CRD420251272759. The study protocol is available in Supplementary File S1. The research was conducted in accordance with the PRISMA Statement guidelines provided in Supplementary Files S2 and S3. The systematic review was evaluated using the JBI Checklist contained in Supplementary File S4.

### 2.1. Search Strategy and Selection Criteria

In December 2025, a search of medical databases was performed, including PubMed, Scopus, Web of Science, and Embase. The search strategy was based on the PICOS framework provided in Supplementary File S5, which guided the overall strategy and keyword selection. A detailed search strategy for each database is available in the Supplementary File S6. Initially, a total of 114 articles were identified. After applying filters, 80 remained. Preliminary screening was conducted using Rayyan [18]. A total of 65 duplicates were automatically detected, of which 42 were removed. Subsequently, two independent researchers conducted a preliminary review of 38 articles based on titles, abstracts, and keywords. A total of 27 articles were excluded, with reasons provided. Articles could be excluded based on more than one category. In the next stage, the full texts of the remaining 11 articles were assessed, from which 4 were qualified for the review. Conflicts were resolved by a third independent researcher. Inter-rater reliability, assessed via Cohen’s kappa test, was 0.732, indicating substantial agreement [19]. Additionally, during the bibliography review, one more article was found and qualified for the study. Ultimately, 5 articles were included in the review. The detailed workflow is presented in Supplementary File S7.

### 2.2. Inclusion and Exclusion Criteria

The inclusion and exclusion criteria, as well as the detailed data extracted from the studies, are presented in Supplementary File S1. Inclusion criteria were original articles, presence of an abstract, published within the last 10 years, and thematically related to nanoplastics, microplastics, and stroke. To obtain as many relevant studies as possible, it was decided to include both prospective and retrospective studies. Exclusion criteria included pediatric population, systematic reviews, meta-analyses, congress abstracts, conference abstracts, and other forms of scientific publications that are not original articles.

### 2.3. Data Extraction

The following data were extracted by three analysts from the selected articles. Based on the common criteria identified during phase 1 of data extraction, the following items were collected:

1. General characteristics: Authors, publication date, country, journal, Impact Factor, number of citations, source of funding, and keywords.
2. Study design and population: Type of study, sample size, sex distribution, mean/median age, and anatomical location of sampling.
3. Methodology: Significance of microplastics for the aim of the study, experimental procedure or exposure assessment, and method of microplastic detection.
4. Microplastic characteristics: Polymer types detected, particle size, and additional findings.
5. Outcomes: Main study results, conclusions, and study limitations.

Due to the methodological heterogeneity of the included studies, a meta-analysis was not performed. A narrative synthesis of the results was conducted instead.

### 2.4. Risk of Bias Assessment

The methodological quality and risk of bias in the included studies were assessed by two independent reviewers. Disagreements were resolved by consensus or consultation with a third reviewer. The following tools were applied based on the study design: ROBINS-E [20] was used for observational human studies, and SYRCLE’s RoB tool [21] was applied to in vivo animal studies. Results were presented using traffic light plots.

## 3. Results

### 3.1. Characteristics of Included Studies

**Figure 1:**
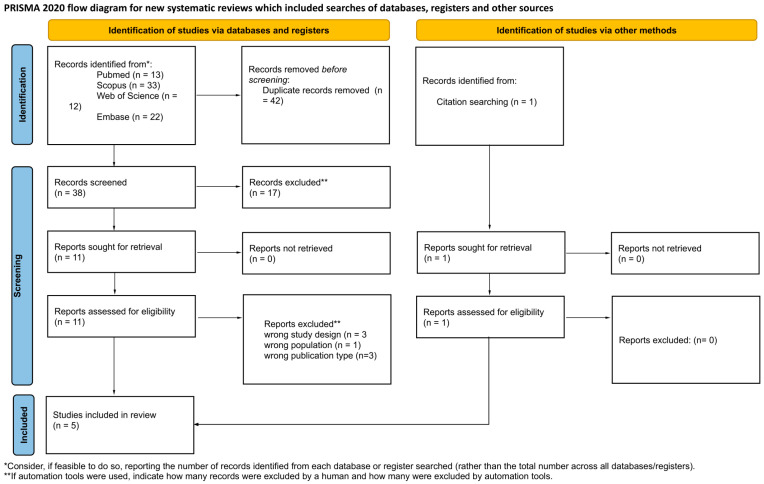
PRISMA 2020 flow diagram illustrating the study selection process for the systematic review.

The process of identifying and selecting scientific evidence was conducted according to PRISMA 2020 guidelines (Supplementary File S2). The detailed selection flow is depicted in Figure 1. Ultimately, five studies meeting all inclusion criteria and providing valuable data for synthesis were included in the systematic review. The included works are characterized by diverse methodologies, encompassing both human observational studies [6,22] and experimental animal models [13,23,24]. Key characteristics and main findings of the included studies are summarized in Table 1. Comprehensive details regarding study methodology, patient demographics, and full quantitative data are provided in Supplementary File S7.

The analysis included observational studies, one cross-sectional [22] and one cohort [6], conducted in China [22] and Italy [6]. These studies involved a total population of 287 patients (30 in the study by Wang et al. [22] and 257 in Marfella et al. [6]) who underwent invasive procedures. The primary subjects of analysis were cardiovascular tissues: thrombi collected from patients with myocardial infarction, stroke, and deep vein thrombosis [22], and atherosclerotic plaques from carotid arteries [6]. Both studies confirmed the presence of MNPs in the examined tissues. Three included in vivo experimental studies [13,23,24] conducted on animal models: two on mice [13,23] and one on rats [24]. These works aimed to assess the impact of micro- and nanoplastic exposure on the nervous and vascular systems. Research models included cerebral ischemia [24] and internal carotid artery occlusion [23]. Studies demonstrated that microplastics can lead to microembolization [13], BBB damage, increased inflammation [24], and neurobehavioral deficits [23].

### 3.2. Methodological Quality of Included Studies

The comprehensive results of the risk-of-bias assessment are presented below, divided by study type.

### 3.3. Observational Studies (ROBINS-E)

**Figure 2:**
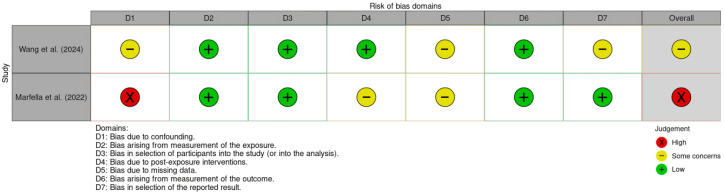
Traffic light plot for human observational studies assessed using the ROBINS-E tool [6,22].

The risk-of-bias visualization for human studies is shown in Figure 2. The study by Marfella et al. [6] was rated as having a high risk of bias, primarily due to confounding factors, specifically the difficulty in isolating the impact of microplastics from other variables such as socioeconomic status or smoking. Issues regarding selective data presentation were also noted. The study by Wang et al. [22] was classified as having some concerns, with primary limitations involving missing data, irregularities in the selection of reported results, and confounding factors.

### 3.4. Experimental Studies (SYRCLE’s RoB)

**Figure 3:**
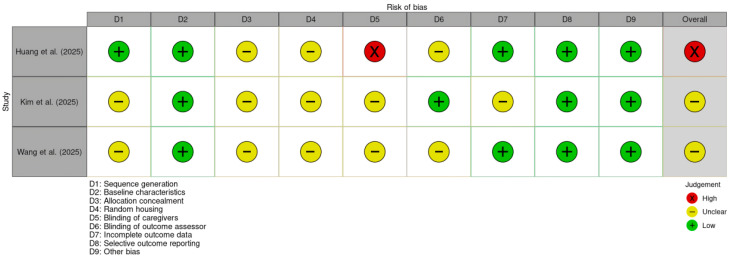
Traffic light plot for animal in vivo studies assessed using the SYRCLE risk-of-bias tool [13,23,24].

Results for animal models are shown in Figure 3. The study by Huang et al. [13] was rated as high risk, as technical personnel were aware of the animals’ group assignments. The remaining works [23,24] raised some concerns; common issues included a lack of detailed description regarding the random allocation technique, lack of clarification on whether investigators knew the allocation sequence, and no information on cage placement strategies to ensure identical environmental conditions.

## 4. Synthesis of Results

### 4.1. Clinical–Epidemiological Results: Presence of Microplastics in the Vascular System and Clinical Risk

#### 4.1.1. Detectability and Composition

Clinical studies indicate that MNPs are detected in both thrombi and atherosclerotic plaques of major arteries [6,22]. Wang et al. [22] identified microplastics in 80% (24/30) of thrombi from patients with ischemic stroke, myocardial infarction, and deep vein thrombosis. Median concentrations were 61.75 μg/g for ischemic stroke, 141.80 μg/g for myocardial infarction, and 69.62 μg/g for deep vein thrombosis. Dominant polymers included poly(hexamethylene adipamide) (PA66), polyvinyl chloride (PVC), and polyethylene (PE); higher concentrations correlated with increased disease severity and D-dimer levels [22]. In a cohort of 304 patients undergoing carotid endarterectomy, MNPs were found in atherosclerotic plaques [6]. PE was detected in approximately 58% of patients (average 21.7 ± 24.5 μg/mg of tissue), while PVC was found in approximately 12%. These particles were often irregular and located within foam cells and the necrotic lipid core of the plaque. This consistently indicates that widely used industrial plastics can enter the body and deposit in cardiovascular structures.

#### 4.1.2. Clinical Risk

The strongest clinical risk data come from Marfella et al. [6]. The risk of a composite endpoint (myocardial infarction, stroke, all-cause mortality) over a 34-month observation was significantly higher in patients with MNPs in their plaques compared to those without [6]. The presence of MNPs independently predicted cardiovascular events, with a hazard ratio of 4.53 (95% CI: 2.00–10.26; *p* < 0.001) after adjusting for traditional risk factors [6]. This suggests that plastic particles in plaques are not just passive markers of exposure but are associated with a significantly higher risk of hard endpoints.

### 4.2. Experimental Results

#### 4.2.1. Circulatory Obstruction and Physical Effect

Huang et al. [13] used miniature two-photon microscopy in vivo to image cortical capillaries in mice after intravenous administration of fluorescently labeled microplastics. They demonstrated that circulating microplastics are rapidly phagocytosed by blood cells, which then become mechanically trapped in cortical capillaries. This leads to cellular microemboli, resulting in chronic impairment of blood flow and subsequent neurological and behavioral abnormalities [13]. A key conclusion of this work is a proposed new mechanism of toxicity: tissue dysfunction results primarily from the indirect effect of cellular emboli and local microcirculatory impairment rather than direct particle penetration into the brain parenchyma [13]. This model aligns with clinical observations where MNPs are detected in thrombi and plaques and correlate with markers of coagulation activation [6,22].

#### 4.2.2. Aggravation of Ischemic Injury and Inflammation

In a global cerebral ischemia (GCI) model, Kim et al. [24] evaluated the effect of oral exposure to polystyrene microplastics on hippocampal damage. Compared to the non-exposed group, the GCI-MP group showed higher numbers of degenerating neurons, intensified inflammatory response, more pronounced microtubule and myelin damage, and poorer neurological and cognitive test results [24]. The authors emphasize that while ischemia remains the dominant factor, the presence of MNPs significantly exacerbates its structural and functional consequences, likely by reinforcing neuroinflammation, cytoskeleton disruption, and demyelination [24]. This suggests that individuals chronically exposed to microplastics may be susceptible to more extensive brain damage and a poorer functional outcome following incidents such as stroke or global ischemia.

### 4.3. Nanoplastics and Chronic Vascular Disease

Wang et al. [23] used a mouse model with chronic internal carotid artery occlusion to assess the impact of intravenous nanoplastic exposure under existing vascular disease conditions [23]. Nanoplastics were found to distribute to the brain and peripheral organs, with marked accumulation in tissues affected by chronic flow impairment. Animals exposed to nanoplastics exhibited significant behavioral disturbances, including exacerbated depressive-like and anxiety-like behaviors, indicating functional consequences of nanoplastic deposition in the brain [23]. The application of proteomic and metabolomic analyses revealed profound alterations in pathways associated with the inflammatory response, oxidative stress, lipid metabolism, and mitochondrial function [23]. These changes were more pronounced in animals with chronic internal carotid artery occlusion, suggesting that vascular disease increases the susceptibility of neural tissue to the toxic effects of nanoplastics. Additionally, it was demonstrated that nanoplastic exposure accelerated the atherosclerotic process, linking nanoplastic toxicity to both brain injury and the progression of vascular disease [23].

### 4.4. Summary of Synthesis Results

The gathered data from human studies and animal experimental models create a consistent picture indicating that the presence of micro- and nanoplastics in the vascular system may be associated with an increased risk of cardiovascular events, including stroke. The phenomena observed in vivo, including microemboli in microcirculation, exacerbated ischemic brain injury, and inflammation, provide a plausible biological explanation for this association [6,13,22,23,24]. However, it should be emphasized that the available data are subject to a degree of uncertainty resulting from the assessed risk of bias, which necessitates caution in interpreting the effect size. This body of evidence provides a foundation for further discussion regarding the potential role of MNPs as a novel risk factor for stroke and other cerebrovascular diseases.

## 5. Discussion

This systematic review provides the first synthesized evidence linking MNPs exposure to the risk and pathology of stroke. By integrating data from human observational studies and in vivo animal models, a coherent narrative emerges: MNPs are not merely passive environmental contaminants but actively accumulate in pathological vascular structures [6,22], potentially exacerbating ischemic injury and increasing the risk of adverse cardiovascular events [6,24]. The detection of MNPs within human thrombi and carotid plaques represents a critical finding. The identified polymers, primarily PE and PVC, mirror the most common plastics used in industrial production, confirming systemic absorption and retention in the human body [6,22].

In contrast, experimental studies included in this review predominantly employ PS particles [13,23,24], despite this polymer not being the most frequently detected in humans. This discrepancy represents a significant limitation, as the biological mechanisms driven by PS in animal models may not fully reflect the pathophysiology of PE and PVC accumulation observed in human atheromas. Additionally, it should be acknowledged that the presence of MNPs in vascular tissues may also reflect cumulative environmental exposures or advanced vascular disease, rather than a direct causal role in stroke pathogenesis. Unlike previous studies identifying MNPs in stool, placenta, or lung tissue [8,9,10], the presence of these particles in high-flow vascular beds suggests they may become embedded in the vessel wall or form a clot matrix. Crucially, the available clinical data suggest that this accumulation is not benign. The strong association between plaque MNP burden and major adverse cardiovascular events indicates that plastic deposits may contribute to plaque instability or rupture, rather than acting solely as innocent bystanders [6]. This aligns with the foreign body hypothesis, which posits that chronic irritation by persistent particles promotes local inflammation and prothrombotic states [6]. Experimental models included in this review bridge the gap between association and causation, offering three distinct mechanisms for MNP-induced vascular harm depending on particle size. First, microplastics (>1 μm) appear to act primarily through physical obstruction; circulating MNPs can be phagocytosed by immune cells, forming cellular microemboli that mechanically block cortical capillaries [13]. This microthrombotic effect explains the perfusion deficits observed even in the absence of large-vessel occlusion [13]. Second, nanoplastics (<1 μm) possess the ability to penetrate the BBB and disrupt intracellular molecular pathways related to oxidative stress and metabolism, particularly in vessels already compromised by chronic disease [23]. Third, MNPs appear to induce a state of inflammatory priming. Chronic exposure sensitizes the brain to ischemic injury, resulting in larger infarct volumes and worse neurological deficits following stroke [24]. Together, these mechanisms suggest that MNP exposure acts as a second hit, rendering the neurovascular unit more vulnerable to acute ischemic events [23,24]. It should be noted that across the experimental studies included in this review, the applied doses and exposure routes may not accurately reflect realistic, chronic low-dose human environmental exposure scenarios. Additionally, studies [13,23,24] specifically employ polystyrene particles, which limits the relevance of their findings to human exposure, where PE and PVC are dominant polymers. Despite these significant insights, the current evidence base has notable limitations. The total number of studies is small, and the human cohorts are drawn from highly selected populations undergoing surgical interventions, limiting generalizability to the broader public [6,22]. It is important to note that MNPs have also been detected in individuals without clinical stroke and in patients with transient ischemic attack, indicating that their presence is not specific to overt cerebrovascular events. Methodological heterogeneity remains a challenge; differences in detection techniques (e.g., pyrolysis–GC/MS used in [22] versus other methods) complicate the direct comparison of particle concentrations across studies. Furthermore, the risk-of-bias assessment revealed concerns regarding confounding factors in observational studies and blinding in animal experiments. Specifically, the high hazard ratios observed in clinical cohorts must be interpreted with caution, as residual confounders could inflate the estimated risk [6]. These limitations highlight the need for experimental models to be better aligned with human-relevant polymers and exposure scenarios. Taken together, the issues discussed above suggest that MNP burden may currently be interpreted as a marker of environmental exposure or disease severity rather than definitive evidence of a causal role in stroke. This review nevertheless supports the hypothesis that MNPs may represent a potential novel, modifiable risk factor for stroke, pending confirmation in longitudinal human studies. Future research must focus on standardized quantification methods to establish dose–response relationships and large-scale longitudinal studies to validate the predictive value of MNP burden. From a public health perspective, the findings support the urgent need for strategies to mitigate plastic pollution. If confirmed, the link between environmental plastic exposure and cerebrovascular disease could redefine prevention guidelines, adding environmental health to the traditional pillars of stroke prevention.

An additional limitation of this review is the relatively small number of studies meeting the inclusion criteria, which reflects the emerging and pioneering nature of this research field. Due to the novelty of investigating the link between micro- and nanoplastic exposure and cerebrovascular disease, the available literature remains limited. Strict inclusion criteria were applied to ensure methodological rigor, leading to the exclusion of studies with insufficient quality or unclear outcome definitions. Consequently, only five studies were included, representing a balance between scientific validity and completeness rather than a lack of available evidence.

## 6. Conclusions

This systematic review, comprising an analysis of five studies, provides preliminary but consistent evidence suggesting that MNPs may constitute a new, potentially modifiable risk factor for stroke and cardiovascular diseases. The analysis of available research clearly confirms the presence of MNPs, particularly polyethylene and polyvinyl chloride, in key human pathological structures of the circulatory system, such as atherosclerotic plaques and thrombi collected from ischemic stroke patients. Significantly, clinical observations indicate a strong association between the presence of these contaminants in tissues and a markedly increased risk of myocardial infarction, stroke, or death. This association is biologically supported by in vivo experimental studies, which have shown that MNPs can induce the formation of cellular microemboli, intensify inflammation, and exacerbate ischemic brain injury.

However, it must be emphasized that the current state of knowledge is based on a limited number of studies with varying methodological quality and a high risk of bias in certain domains. Importantly, MNPs detected in vascular tissues may represent markers of cumulative environmental exposure and disease severity rather than a direct causal factor in cerebrovascular pathology. Therefore, although these results are alarming and justify treating plastic pollution as a public health problem, establishing a definitive causal link requires further research in larger populations using standardized detection methods. Future work should focus on elucidating the molecular mechanisms of MNPs’ interaction with the coagulation system and assessing whether the reduction in environmental exposure can realistically decrease the risk of stroke.

## Figures and Tables

**Table 1:** Characteristics and main findings of included studies.

Study	Population/Model	Detection Method	MNP Characteristics (Type, Size)	Key Findings and Clinical Implications	Main Limitations
Wang et al. (2024) (Observational) [22]	Patients (*n* = 30: CVD); mean age, 65.2 y	Py-GC/MS, LDIR, SEM	PA 66, PE, PVC, PS, PET, PMMA, PC, PA6, PP, PBAT; 200–500 μm	MNPs detected in 80% of thrombi; higher MNP concentrations correlated with elevated D-dimer levels, suggesting a link to thrombosis severity	Small sample size
Marfella et al. (2024) (Prospective Cohort) [6]	Patients (*n* = 257) with asymptomatic carotid stenosis; age range: 18–75 y	Py-GC/MS, EM, stable isotope analysis	PE, PVC; jagged particles <1 μm	MNPs in plaque associated with increased risk of MI, stroke, or death (HR 4.53); linked to elevated inflammatory markers	Risk of laboratory contamination
Huang et al. (2025) (Exp. In vivo) [13]	Mice (*n* = 5 per group); male wild-type C57BL/6J; age range: 8–10 wk	mTPM, FACS, immunofluorescence staining, LSCI	PS; 5 μm, 2 μm, 0.08 μm	Phagocytosis of circulating MNPs by immune cells leads to capillary obstruction and reduced cerebral blood flow	Animal model specificity
Kim et al. (2025) (Exp. In vivo) [24]	Rats (*n* = 5–9 per group); male Sprague Dawley; 8 wk	FJB Staining, immunofluorescence, Western blot, MWM, mNSS	PS; 0.5 μm	Oral exposure to MNPs prior to ischemia exacerbates brain injury, increases hippocampal neuronal death, and worsens neuroinflammation compared to controls	High experimental dosage
Wang et al. (2025) (Exp. In vivo) [23]	Mice (*n* = 8 per group); male C57BL/6J; 8 wk	TEM, confocal microscopy, LSCI, LC-MS/MS	PS-NPs; 50 nm	Chronic exposure leads to NP accumulation in the brain, inducing anxiety-like behavior and promoting atherosclerosis through a Th17/Treg immune imbalance	Short experimental duration

Abbreviations: CVD: cardiovascular disease; EM: electron microscopy; FACS: fluorescence-activated cell sorting; FJB: Fluoro-Jade B; HR: hazard ratio; LSCI: laser speckle contrast imaging; LDIR: laser direct infrared; MNPs: micro- and nanoplastics; mTPM: miniature two-photon microscopy; mNSS: modified neurological severity score; MWM: Morris water maze; PE: polyethylene; PS: polystyrene; PVC: polyvinyl chloride; Py-GC/MS: pyrolysis–-gas chromatography/mass spectrometry; SEM: scanning electron microscopy; TEM: transmission electron microscopy.

## Data Availability

No new data were created or analyzed in this study. Data sharing is not applicable to this article.

## Supplementary Materials

The following supporting information can be downloaded at https://www.mdpi.com/article/10.3390/jox16010034/s1: File S1: Study Protocol; File S2: PRISMA 2020 Checklist; File S3: PRISMA 2020 Abstract Checklist; File S4: JBI Critical Appraisal Checklist; File S5: PICOS Framework; File S6: Detailed Search Strategy; File S7: Detailed Characteristics of Included Studies. References [6,13,22,23,24] are cited in the Supplementary Materials.

## Abbreviations

BBB	Blood–Brain Barrier
CVD	Cardiovascular Disease
EM	Electron Microscopy
GCI	Global Cerebral Ischemia
LDIR	Laser Direct Infrared Spectroscopy
LSCI	Laser Speckle Contrast Imaging
MNPs	Micro- and Nanoplastics
MPs	Microplastics
mNSS	Modified Neurological Severity Score
mTPM	Miniature Two-Photon Microscopy
MWM	Morris Water Maze
NPs	Nanoplastics
PA66	Polyamide 66
PE	Polyethylene
PRISMA	Preferred Reporting Items for Systematic Reviews and Meta-Analyses
PS	Polystyrene
PVC	Polyvinyl Chloride
Py-GC/MS	Pyrolysis–Gas Chromatography/Mass Spectrometry
RoB	Risk of Bias
ROBINS-E	Risk Of Bias In Non-randomized Studies—of Exposures
SEM	Scanning Electron Microscopy
SYRCLE	Systematic Review Centre for Laboratory Animal Experimentation
TEM	Transmission Electron Microscopy

## References

[1] Katan M., Luft A. (2018). Global Burden of Stroke. Semin. Neurol..

[2] Feigin V.L., Brainin M., Norrving B., O Martins S., Pandian J., Lindsay P., Grupper M.F., Rautalin I. (2025). World Stroke Organization: Global Stroke Fact Sheet 2025. Int. J. Stroke.

[3] Sun T., Teng Y., Ji C., Li F., Shan X., Wu H. (2024). Global prevalence of microplastics in tap water systems: Abundance, characteristics, drivers and knowledge gaps. Sci. Total Environ..

[4] Kadac-Czapska K., Knez E., Grembecka M. (2024). Food and human safety: The impact of microplastics. Crit. Rev. Food Sci. Nutr..

[5] Sewwandi M., Wijesekara H., Rajapaksha A.U., Soysa S., Vithanage M. (2023). Microplastics and plastics-associated contaminants in food and beverages; Global trends, concentrations, and human exposure. Environ. Pollut..

[6] Marfella R., Prattichizzo F., Sardu C., Fulgenzi G., Graciotti L., Spadoni T., D’oNofrio N., Scisciola L., La Grotta R., Frigé C. (2024). Microplastics and Nanoplastics in Atheromas and Cardiovascular Events. N. Engl. J. Med..

[7] Zhao B., Rehati P., Yang Z., Cai Z., Guo C., Li Y. (2024). The potential toxicity of microplastics on human health. Sci. Total Environ..

[8] Iannuzzi G.L., D’alto M., Bosso G., Montella A.P., D’oria V., Pellegrino L., Boccaforno G., Masi A., Orlando A., Franco R. (2025). Microplastics, Nanoplastics and Heart Contamination: The Hidden Threat. J. Clin. Med..

[9] Braun T., Ehrlich L., Henrich W., Koeppel S., Lomako I., Schwabl P., Liebmann B. (2021). Detection of Microplastic in Human Placenta and Meconium in a Clinical Setting. Pharmaceutics.

[10] Zhang T., Liao Y., Ling J., Zhang J., Zhang D., Yin X., Yu P., Liu X. (2025). Tiny trouble: Microplastics, nanoplastics, and their heartfelt impact on cardiovascular health. Cardiovasc. Res..

[11] Hirt N., Body-Malapel M. (2020). Immunotoxicity and intestinal effects of nano- and microplastics: A review of the literature. Part. Fibre Toxicol..

[12] Lee Y.H., Zheng C.M., Wang Y.J., Wang Y.L., Chiu H.W. (2025). Effects of microplastics and nanoplastics on the kidney and cardiovascular system. Nat. Rev. Nephrol..

[13] Huang H., Hou J., Li M., Wei F., Liao Y., Xi B. (2025). Microplastics in the bloodstream can induce cerebral thrombosis by causing cell obstruction and lead to neurobehavioral abnormalities. Sci. Adv..

[14] Makwana B., Khadke S., Kumar A., Nasir K., Wadhera R., Shah R., Sheth S., Kong Y., Navas-Acien A., Adamkiewicz G. (2025). Marine Microplastic Levels and the Prevalence of Cardiometabolic Diseases in US Coastline Counties. J. Am. Heart Assoc..

[15] Kutralam-Muniasamy G., Shruti V.C., Pérez-Guevara F., Roy P.D. (2023). Microplastic diagnostics in humans: “The 3Ps” Progress, problems, and prospects. Sci. Total Environ..

[16] Barceló D., Picó Y., Alfarhan A.H. (2023). Microplastics: Detection in human samples, cell line studies, and health impacts. Environ. Toxicol. Pharmacol..

[17] Yuan Z., Nag R., Cummins E. (2022). Human health concerns regarding microplastics in the aquatic environment—From marine to food systems. Sci. Total Environ..

[18] Ouzzani M., Hammady H., Fedorowicz Z., Elmagarmid A. (2016). Rayyan-a web and mobile app for systematic reviews. Syst. Rev..

[19] McHugh M.L. (2012). Interrater reliability: The kappa statistic. Biochem. Med..

[20] Higgins J.P.T., Morgan R.L., Rooney A.A., Taylor K.W., Thayer K.A., Silva R.A., Lemeris C., Akl E.A., Bateson T.F., Berkman N.D. (2024). A tool to assess risk of bias in non-randomized follow-up studies of exposure effects (ROBINS-E). Environ. Int..

[21] Hooijmans C.R., Rovers M.M., de Vries R.B., Leenaars M., Ritskes-Hoitinga M., Langendam M.W. (2014). SYRCLE’s risk of bias tool for animal studies. BMC Med. Res. Methodol..

[22] Wang T., Yi Z., Liu X., Cai Y., Huang X., Fang J., Shen R., Lu W., Xiao Y., Zhuang W. (2024). Multimodal detection and analysis of microplastics in human thrombi from multiple anatomically distinct sites. eBioMedicine.

[23] Wang L., Ma J.-Q., Song L.-J., Qu X.-P., Zhang Y., Fan H.-M., Wang C., Zheng L.-L., Gao G.-D., Qu Y. (2025). Comprehensive multi-omics, behavioral and morphological analysis of the hazards of nano-plastics in mice with internal carotid artery occlusion. Ecotoxicol. Environ. Saf..

[24] Kim D.Y., Park M.K., Yang H.W., Woo S.Y., Jung H.H., Son D.-S., Choi B.Y., Suh S.W. (2025). Effects of Microplastic Accumulation on Neuronal Death After Global Cerebral Ischemia. Cells.

