# Supplementary material for: Micro- and Nanoplastics as a Potential Risk Factor for Stroke: A Systematic Review
Source: J Xenobiot. 2026 Feb 14;16(1):34. doi: 10.3390/jox16010034 (PMC12922052; doi:10.3390/jox16010034)
Supplement: Supplementary file 1 [file jox-16-00034-s001.zip › File_S1_Study_Protocol.pdf]

Study protocol

Article title: Micro- and Nanoplastics as a Potential Risk Factor for Stroke: A Systematic Review

Objective: To systematically assess the potential role of micro- and nanoplastics (MNPs) as an emerging risk factor for cardiovascular diseases. This review synthesizes evidence regarding the accumulation of MNPs in human vascular tissues and their potential pathophysiological mechanisms leading to thrombosis and cerebral ischemia based on both clinical and experimental studies.

Methods:

1. **Search Strategy and Selection Criteria**

A comprehensive study was conducted using various databases such as MEDLINE via PubMed, Scopus, Web of Science and Embase. The search strategy was based on the PICOS scheme, through which the entire search strategy was developed and the search terms selected:
Terms: microplastics, nanoplastics, Strokes, Cerebral Stroke, Brain Vascular Accident, Acute Stroke

2. **Inclusion and exclusion criteria**

Inclusion criteria for articles used in the study were: original articles, having an abstract, from the last 10 years and relating to nanoplastic, and thematically related to stroke.

To obtain as many studies meeting the other criteria as possible, it was decided to include both prospective and retrospective studies in the systematic review.

Exclusion criteria for articles used in the study included: paediatric population, systematic reviews, meta-analyses, congress abstracts, conference abstracts and other forms of scientific publications that are not original articles. All studies older than 10 years.

3. **Study selection**

The process of selection of the studies of interest was conducted by two reviewers. After removing duplicates (n=65), they independently screened abstracts to select potential eligible studies. Then, full-text reports were analyzed for eligibility. A third reviewer resolved possible discrepancies highlighted during the selection process, if consensus was not found. In addition to the systematic database search, the reference lists of key review articles and included studies were manually searched to identify relevant primary research not captured by the initial strategy. Ultimately, a total of 5 articles (4 from the database search and 1 from the manual search) were included in the study.

4. **Data Extraction**

The following data were extracted by three analysts (MK, JJ, ZP) from the selected articles. According to the common criteria highlighted during phase 1 of the data extraction, the following items were extracted:

- **General characteristics:** Authors, publication date, country, journal, Impact Factor (IF), number of citations (Scopus), source of funding, and keywords.
- **Study design and population:** Type of study, sample size (number of patients or animals), sex distribution (male/female), mean/median age, and anatomic location of sampling.
- **Methodology:** Significance of microplastics in the study aim, experimental procedure or exposure assessment ("what was done"), and method of microplastic detection (e.g., Py-GC/MS, LDIR, fluorescence).
- **Microplastic characteristics:** Polymer types detected, particle size, and additional findings (e.g., adsorbed contaminants).
- **Outcomes:** Main study results, conclusions, and study limitations.

5. **Checklists**

a. PRISMA 2020 checklist
b. PRISMA 2020 abstract checklist
c. JBI Checklist
d. PICOS

6. **Risk of bias assessment**

The methodological quality and risk of bias in the included studies were assessed by two independent reviewers. Disagreements were resolved by consensus or consultation with a third reviewer. The following tools were applied based on the study design:

- ROBINS-E (Risk Of Bias In Non-randomized Studies - of Exposures) was used for observational human studies.
- SYRCLE's RoB tool was applied to in vivo animal studies.

Results are presented using summary tables and traffic light plots.
