# Supplementary material for: Micro- and Nanoplastics as a Potential Risk Factor for Stroke: A Systematic Review
Source: J Xenobiot. 2026 Feb 14;16(1):34. doi: 10.3390/jox16010034 (PMC12922052; doi:10.3390/jox16010034)
Supplement: Supplementary file 1 [file jox-16-00034-s001.zip › File_S4_JBI_Checklist.pdf]

JBI Critical Appraisal Checklist for Systematic Reviews and Research Syntheses

	Yes	No	Unclear	Not applicable
1. Is the review question clearly and explicitly stated?	X	☐	☐	☐
2. Were the inclusion criteria appropriate for the review question?	X	☐	☐	☐
3. Was the search strategy appropriate?	X	☐	☐	☐
4. Were the sources and resources used to search for studies adequate?	X	☐	☐	☐
5. Were the criteria for appraising studies appropriate?	X	☐	☐	☐
6. Was critical appraisal conducted by two or more reviewers independently?	X	☐	☐	☐
7. Were there methods to minimize errors in data extraction?	X	☐	☐	☐
8. Were the methods used to combine studies appropriate?	X	☐	☐	☐
9. Was the likelihood of publication bias assessed?	☐	☐	☐	X
10. Were recommendations for policy and/or practice supported by the reported data?	X	☐	☐	☐
11. Were the specific directives for new research appropriate?	X	☐	☐	☐

Overall appraisal: Include X Exclude ☐ Seek further info ☐

Comments (Including reason for exclusion)
