# Supplementary material for: Micro- and Nanoplastics as a Potential Risk Factor for Stroke: A Systematic Review
Source: J Xenobiot. 2026 Feb 14;16(1):34. doi: 10.3390/jox16010034 (PMC12922052; doi:10.3390/jox16010034)
Supplement: Supplementary file 1 [file jox-16-00034-s001.zip › File_S5_PICOS_Framework.pdf]

PICO scheme of searching

PICO elements	Keywords	Search terms	Search strategy
**P** (**P**atient or/and **P**opulation)	Adult patients with stroke.<br>Animals used in stroke/ischemia research.	Stroke	Strokes OR Cerebral Stroke OR Brain Vascular Accident OR Acute Stroke
**I** (**I**ntervention)	Presence of microplastic in the results.	Microplastic	microplastics OR nanoplastics
**C** (**C**omparison)	not applicable		
**O** (**O**utcome)	not applicable.	not applicable	not applicable
