# Supplementary material for: Micro- and Nanoplastics as a Potential Risk Factor for Stroke: A Systematic Review
Source: J Xenobiot. 2026 Feb 14;16(1):34. doi: 10.3390/jox16010034 (PMC12922052; doi:10.3390/jox16010034)
Supplement: Supplementary file 1 [file jox-16-00034-s001.zip › File_S6_Search_Strategy.pdf]

Search strategy (24.12.2025)

String:

(microplastics OR nanoplastics) AND (Strokes OR Cerebral Stroke OR Brain Vascular Accident OR Acute Stroke)

PubMed:

(
"Microplastics"[MeSH Terms] OR microplastic*[Title/Abstract] OR
nanoplastic*[Title/Abstract] OR "plastic particle*"[Title/Abstract]
)
AND
(
"Stroke"[MeSH Terms] OR "Cerebrovascular Disorders"[MeSH Terms] OR
"Brain Ischemia"[MeSH Terms] OR "Intracranial Hemorrhages"[MeSH Terms] OR
stroke*[Title/Abstract] OR "cerebrovascular accident*"[Title/Abstract] OR
"brain ischemia"[Title/Abstract] OR "intracerebral hemorrhag*"[Title/Abstract] OR
"haemorrhag*"[Title/Abstract]
)

22 -> Filters: Abstract, full text, in the last 10 years -> 13

Scopus:

TITLE-ABS-KEY ((microplastic* OR "micro plastic*" OR nanoplastic* OR "nano plastic*" OR "plastic particle*" OR "plastic debris") AND (stroke* OR "cerebral stroke*" OR "brain vascular accident*" OR "cerebrovascular accident*" OR "acute stroke*" OR "ischemic stroke*" OR "ischaemic stroke*" OR "hemorrhagic stroke*" OR "haemorrhagic stroke*" OR "intracerebral hemorrhag*" OR "intracerebral haemorrhag*" OR "brain ischemia" OR "cerebral ischemia"))

33 -> Filters: in the last 10 years, article -> 33

Web of Science:

TS=(
(microplastic* OR "micro plastic*" OR nanoplastic* OR "nano plastic*" OR "plastic particle*" OR "plastic debris")
AND
(stroke* OR "cerebral stroke*" OR "cerebrovascular accident*" OR "brain vascular accident*" OR "acute stroke*" OR
"ischemic stroke*" OR "ischaemic stroke*" OR "hemorrhagic stroke*" OR "haemorrhagic stroke*" OR
"intracerebral hemorrhag*" OR "intracerebral haemorrhag*" OR "brain ischemia" OR "cerebral ischemia")
)

19 -> Filters: in the last 10 years, article -> 12

Embase:

(
'microplastic'/exp OR 'nanoplastic'/exp OR 'plastic particle'/exp OR
(microplastic* OR "micro plastic*" OR nanoplastic* OR "nano plastic*" OR
"plastic particle*" OR "plastic debris"):ti,ab,kw

)
AND
(
'cerebrovascular accident'/exp OR 'brain ischemia'/exp OR 'intracerebral hemorrhage'/exp OR
(stroke* OR "cerebral stroke*" OR "cerebrovascular accident*" OR "brain vascular accident*" OR
"acute stroke*" OR "ischemic stroke*" OR "ischaemic stroke*" OR
"hemorrhagic stroke*" OR "haemorrhagic stroke*" OR
"intracerebral hemorrhag*" OR "intracerebral haemorrhag*" OR
"brain ischemia" OR "cerebral ischemia"):ti,ab,kw
)

40 -> Filters: in the last 10 years, article, review, clinical trail -> 22

Results of search summary:

All articles searched: 114
All articles searched after filter: 80
All articles in scanning (Rayyan) before automated duplicates detect: 80
Duplicates automatically detected: 65
Duplicates deleted: 42
First scanning (Rayyan): 38
Full-text articles assessed for eligibility: 11
